# Simultaneous Modified Tibial Plateau Leveling Osteotomy and Tibial Tuberosity Transposition for Grade IV Medial Patellar Luxation and Cranial Cruciate Ligament Disease in Small-Breed Dogs

**DOI:** 10.3390/ani15071042

**Published:** 2025-04-04

**Authors:** Changsu Jung, Byung-Jae Kang

**Affiliations:** 1 JCS Animal Surgical Center, Goyang-si 10380, Republic of Korea; greatchangsu@gmail.com; 2 Department of Veterinary Clinical Sciences, College of Veterinary Medicine and Research Institute for Veterinary Science, Seoul National University, Seoul 08826, Republic of Korea

**Keywords:** medial patellar luxation, cranial cruciate ligament disease, tibial plateau leveling osteotomy, tibial tuberosity transposition, small-breed dogs

## Abstract

This study explored the complications and prognosis of modified tibial plateau leveling osteotomy with tibial tuberosity transposition (mTPLO-TTT) for simultaneously correcting high-grade medial patellar luxation (MPL) and cranial cruciate ligament disease (CCLD) in small-breed dogs. This retrospective study evaluated patient data, lameness scores, radiographic outcomes, and complications over a median follow-up period of 10 weeks. Additionally, an owner interview was conducted 6 months postoperatively. Nine stifles from seven dogs were included in this study. All cases showed satisfactory patellar alignment and stability after surgery, with no major complications or reluxations. The lameness scores improved, and radiographic assessments confirmed implant stability and appropriate bone healing. Owner-reported outcomes at 6 months were also favorable. These findings suggest that simultaneous mTPLO-TTT is an effective surgical option for small-breed dogs with concurrent CCLD and Grade IV MPL.

## Simple Summary

Medial patellar luxation (MPL) and cranial cruciate ligament disease (CCLD) are common causes of hindlimb lameness in dogs. Severe MPL is frequently observed with CCLD in small and toy breeds, rendering surgical correction difficult. Here, we evaluated the feasibility of a combined surgical approach termed modified tibial plateau leveling osteotomy and tibial tuberosity transposition (mTPLO-TTT) for these conditions. We assessed nine cases (seven dogs) postoperatively (at 6–14 weeks). We followed the long-term results through telephone interviews with the owners six months after surgery or later. No major complications were observed, and the postoperative gait recovery and radiographic outcomes were favorable. Owner satisfaction was excellent. Therefore, simultaneous mTPLO-TTT is an effective surgical option for managing severe MPL and CCLD in dogs.

## 1. Introduction

Medial patellar luxation (MPL) and cranial cruciate ligament (CCL) disease (CCLD) are common causes of hindlimb lameness in small-breed dogs [1,2,3,4]. CCLD frequently occurs secondarily to MPL, with a higher incidence in cases of Grade IV MPL [5,6,7,8,9].

When MPL and CCL ruptures coexist, both conditions must be addressed to restore normal stifle joint function. Tibial plateau leveling osteotomy (TPLO) has been modified to repair MPL [10,11,12,13,14]. Two surgical approaches—modified TPLO (mTPLO) [10,11] and TPLO combined with tibial tuberosity transposition (TPLO-TTT) [12,13]—have been introduced to simultaneously address MPL and CCLD.

The mTPLO technique involves medializing the proximal tibial segment to relatively lateralize the tibial tuberosity [10,11]. However, most studies primarily included heavier dogs and the implants used were unsuitable for small breeds. Moreover, patients with Grade IV MPL were excluded from these studies [10,11].

Similarly, the TPLO-TTT technique demonstrated positive overall outcomes [12,13]. However, challenges were noted in small breeds, and cases involving Grade IV MPL were limited [12,13]. Additionally, instances of reluxation were reported in Grade IV MPL cases [12,13].

Despite these advancements, data on TPLO application in small breeds with Grade IV MPL and CCLD remain scarce [10,11,12,13]. Correcting Grade IV MPL is inherently challenging [15,16], and treating Grade IV MPL and CCLD simultaneously in a single surgical procedure is even more difficult. The aforementioned mTPLO and TPLO-TTT techniques are based on the principle of lateral shifting of the tibial tuberosity. Therefore, we hypothesized that combining these two techniques could synergistically enhance their respective effects, potentially providing a surgical approach to address both Grade IV MPL and CCLD simultaneously. This study aimed to evaluate the benefits and limitations of simultaneous mTPLO and tibial tuberosity transposition (mTPLO-TTT) for correcting Grade IV MPL and CCLD.

## 2. Materials and Methods

### 2.1. Case Selection Criteria

Medical records from the JCS Animal Surgical Center database were reviewed to identify dogs undergoing mTPLO-TTT for concurrent CCLD and MPL between 8 November 2023 and 8 November 2024. Each case underwent preoperative, postoperative, and radiological evaluations, with follow-ups conducted 6–14 weeks post-surgery. Nine cases, including two with bilateral corrections, were identified and fit the inclusion criteria. Cases requiring simultaneous distal femoral osteotomy or lateral fabellar suturing were excluded. Informed consent was obtained from all pet owners. All surgeries were performed by a single surgeon (Changsu Jung), who also conducted the preoperative and postoperative evaluations.

### 2.2. Medical Record Review

The collected data included limb involvement, lameness scores (pre- and postoperative), MPL grade, radiographic findings, surgical techniques, implants used, complications, and follow-up outcomes. Lameness was graded on a 0–5 numeric scale: 0 (clinically sound), 1 (barely detectable), 2 (mild), 3 (moderate), 4 (severe), and 5 (non-weight-bearing) [17,18].

### 2.3. Preoperative Planning

Frontal and sagittal radiographs were used to assess bone deformities and plan the TPLO location, tibial tuberosity osteotomy, and implant selection. Surgical planning was performed using digital templating (vPOP Pro; VETSOS Education Ltd., Shrewsbury, UK).

**Figure 1:**
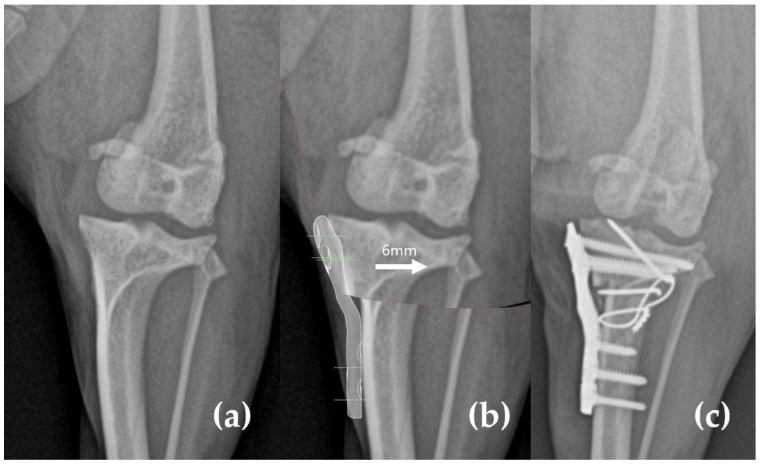
Preoperative surgical planning on the frontal plane radiograph. (**a**) Preoperative radiograph. (**b**) Preoperative planning. The lateral displacement of the tibial tuberosity was calculated. The 2 mm lateral displacement induced by the modified tibial plateau leveling osteotomy (TPLO) plate and additional displacement (indicated by the arrow) required for patellar ligament realignment during tibial tuberosity transposition (TTT) were measured and incorporated into the surgical plan. (**c**) Postoperative radiograph.

Initially, the degree of lateral displacement of the tibial tuberosity was calculated on frontal plane radiographs. The 2 mm lateral displacement induced by the modified TPLO plate, along with additional displacement needed for patellar ligament realignment during TTT, was measured and integrated into the surgical plan (Figure 1).

On the sagittal plane radiograph, the osteotomy was centered on the intercondylar tubercles or articular surface level along the functional tibial axis [19,20]. Three key measurements (D1, D2, and D3) were obtained for surgical guidance to ensure sufficient tibial tuberosity size for reattachment with appropriately sized implants [13]. The preoperative tibial plateau angle (TPA) was calculated as described in prior studies [19,20].

### 2.4. Surgical Technique

Standard preanesthetic and analgesic protocols were followed [21]. Cefazolin (33 mg/kg, IV) was administered as preoperative antibiotic prophylaxis and repeated every 120 min. The dogs were premedicated with butorphanol (0.2 mg/kg, IV) or hydromorphone (0.1 mg/kg, IV) and meloxicam (0.1 mg/kg, IV) for preemptive analgesia. Anesthesia was induced with propofol (6 mg/kg, IV) and maintained with an isoflurane–oxygen mixture. For epidural anesthesia, dogs were positioned in sternal recumbency. A 22-gauge spinal needle was inserted into the lumbosacral space, and 0.5% bupivacaine (1 mg/kg) was injected slowly.

Dogs were positioned in dorsal recumbency, and medial arthrotomy was performed to inspect the CCL and medial meniscus. Meniscal injuries were addressed if present. Lateral arthrotomy, including trochlear block recession, medial retinacular release, and lateral capsular imbrication, was performed as an adjunct MPL correction technique [13].

**Figure 2:**
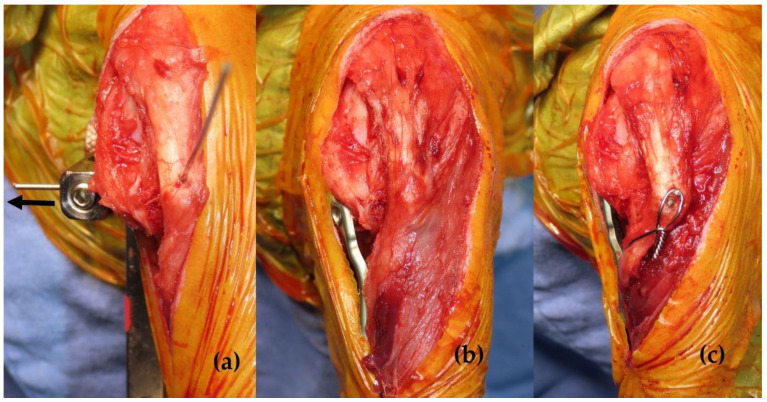
Gross intraoperative image of the medial surface of the stifle joint. (**a**) The proximal tibial segment was rotated to achieve a postoperative tibial plateau angle (TPA) of 5°–6° and translated medially by 2 mm, with the proximal jig pin adjusted to 2 mm medially (indicated by the arrow). (**b**) Fixation was accomplished using a modified 1.5/2.0 mm locking TPLO plate. (**c**) Additionally, the tibial tuberosity was laterally transposed to align the patellar ligament with the trochlear sulcus and secured using two pins and a tension band wire.

TPLO was performed using a jig aligned with the intercondylar tubercles [22]. The proximal tibial segment was rotated to achieve a postoperative TPA of 5°–6° and medially translated to fit the shape and size of the bone with the pre-contoured 2 mm offset modified 1.5/2.0 mm locking TPLO plate (titanium, Jeil Medical Corp., Arix Vet system, Seoul, Republic of Korea) by adjusting the jig accordingly [23]. Fixation was achieved using the TPLO plate and screws (Figure 2a,b).

Tibial tuberosity osteotomy was conducted while preserving the distal cortical continuity. Subsequently, the tuberosity was laterally transposed to align the patellar ligament with the trochlear sulcus and secured using two pins and a tension band wire [12,13] (Figure 2c). At the final stage of the operation, before suturing the subcutaneous tissue, bupivacaine combined with temperature-responsive hydrogel, ez:AP^®^ (TGel Bio Corp., Seoul, Republic of Korea), was injected into the subcutaneous tissue surrounding the incision line using a 1 mL integrated needle [24]. The total bupivacaine dosage injected was dependent on the patient’s body weight and the incision length and did not exceed 2 mg/kg. Based on anesthesia chart records, the surgical duration from skin incision to completion was recorded in 10 min increments. For bilateral procedures, the total operative time was divided in half and recorded accordingly.

**Figure 3:**
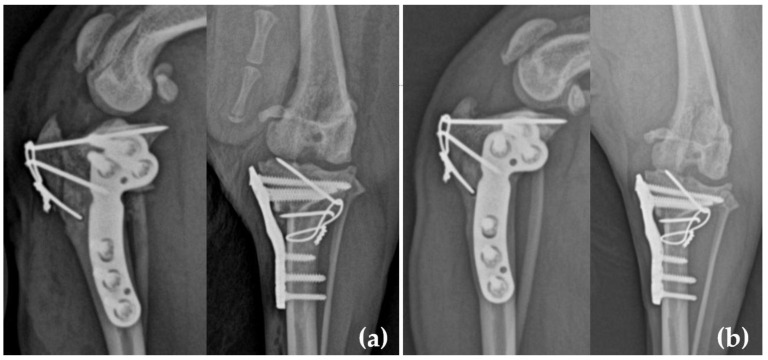
(**a**) Immediate and (**b**) 10-week postoperative radiographs of the stifle after a simultaneous modified TPLO and TTT in dog number 4. In (**a**) the patella located at the center of the trochlear groove and implants securely fixing the osteotomy site under compression are observed. (**b**) A radiographic examination at 10 weeks revealed that the implant position remained stable, and signs of bone union at the osteotomy site were observed.

Postoperative evaluations included a range of motion, limb alignment, and stability assessments. Radiographs confirmed implant positioning and patellar alignment (Figure 3a).

### 2.5. Postoperative Care

After surgery, each patient was given a fentanyl patch for analgesia before discharge, based on body weight. Firocoxib (5 mg/kg once daily) and cefadroxyl (30 mg/kg twice daily) were administered for 5–10 days after hospital discharge. At discharge, an Elizabethan collar was applied, and an adhesive postoperative dressing (Dressing-care^®^, Shinhan Medical Corp., Seoul, Republic of Korea) was placed over the surgical site with the aim of minimizing direct environmental contamination. The dressing was removed 1–3 days postoperatively, and owners were instructed to disinfect the incision site twice daily using a cotton ball soaked with 0.1% chlorhexidine solution. Skin sutures were removed at 14 days.

Postoperative care consisted of 6 weeks of confinement (cage or small room) with short leash walking limited to a maximum of 5 min when necessary. After 6 weeks, the dogs were allowed free walking in the owner’s home and leash walking up to 6 times daily at 10 min per walk, which was gradually increased to 30 min per walk. For owners concerned about gait recovery, a follow-up visit for clinical and radiographic evaluation was scheduled for 4–6 weeks postoperatively. In cases where recovery was deemed satisfactory, the last follow-up was at 10–14 weeks.

### 2.6. Outcome Assessment

Clinical evaluations included lameness scoring and physical examination. Signs of pain, crepitus, and range of motion were documented upon stifle manipulation. Indirect tibial thrust, patellar stability, patellar ligament thickening, and signs of pain on palpation of the surgical site were also recorded. Radiographic assessments were conducted to evaluate the implant positioning, bone healing, and patellar alignment. Additionally, the likelihood of complete healing (stable osteotomy) was rated on a scale of 1–5, following the criteria established in previous studies (Table 1) [19,25]. The complications were classified as major (requiring surgical intervention) or minor (manageable with medical treatment). In addition, owner-assessed outcomes, including activity level, time-to-peak function, and overall results, were evaluated through follow-up phone interviews conducted in accordance with methods outlined in previous studies [26]. The minimum follow-up period for these phone interviews was 6 months.

## 3. Results

### 3.1. Patient Characteristics

Seven dogs (nine stifles) satisfied the inclusion criteria. The median age was 6.67 years (range: 5–8 years), and the median body weight was 4.46 kg (range: 3.1–5.45 kg). Three were spayed females, and four were neutered males. All nine stifles had Grade IV MPL.

### 3.2. Surgical Procedures

All nine stifles underwent mTPLO-TTT. Complete CCL rupture was observed in all cases with no meniscal injuries. Adjunctive MPL correction techniques were performed for all stifles. No intraoperative complications occurred, and radiographs confirmed appropriate postoperative TPA (2.61° ± 1.96°), patellar alignment, and implant positioning. The anesthesia time during surgery ranged from 60 to 90 min, and all patients exhibited smooth recovery from anesthesia.

### 3.3. Outcomes

The median period of the last follow-up visit was 10 weeks (range: 6–12 weeks). Before surgery, all patients exhibited severe lameness (4.11 ± 0.31) and loss of function. At the last postoperative follow-up, most dogs showed near-normal gait (0.44 ± 0.83). In three dogs (five stifles) that underwent follow-up at 4–6 weeks postoperatively, mild lameness (2.0 ± 0) was observed. In seven cases that were re-evaluated at 10–14 weeks, normal gait was restored without evidence of patellar relaxation. No major or minor complications were observed. Radiographic evaluation at 4–6 weeks postoperatively revealed insufficient bone healing (2.6 ± 1.02). However, radiographic evaluations at the last follow-up showed stable implants, a well-aligned patella, and an adequate bone healing score (1.11 ± 0.31, Table 2, Figure 3b). Unilateral TPLO surgery was performed on five dogs, and simultaneous bilateral TPLO surgery was performed on two dogs (4 stifles). No differences in outcomes were observed between the two groups.

All patient owners responded to the telephone interview (n = 7). The median follow-up period for the owner questionnaire was 7.7 months (range: 6–10 months). All owners (7/7) reported that their pets’ activity levels had greatly improved. Regarding the time to peak function, 57% (4/7) responded that it occurred within 1–2 months, whereas 43% (3/7) stated that it required 3–4 months. All owners (100%, 7/7) rated the overall outcome as excellent (Table 3).

## 4. Discussion

This study evaluated the feasibility and outcomes of combined mTPLO-TTT for the treatment of Grade IV MPL and CCLD in small-breed dogs. Despite the small sample size, the results suggest that this approach effectively corrects Grade IV MPL while improving lameness.

No fractures or delayed bone union occurred, supporting the structural stability of this method. Additionally, radiographic bone union assessment scores at 6–14 weeks indicated excellent outcomes. This degree of radiographic bone union recovery is consistent with the findings from other studies evaluating bone union following TPLO or TPLO-TTT [12,27]. No recurrence of patellar luxation was observed. Although long-term follow-up is necessary, several quadriceps alignment shifts in mTPLO-TTT may have helped stabilize the patellar position. While one dog having undergone bilateral mTPLO-TTT exhibited mild lameness during the 6-week follow-up, the degree of gait recovery observed was similar to that reported in other studies evaluating gait following TPLO or TPLO-TTT [13,28]. Hind limb function recovery was incomplete, which may be due to the short duration between the surgery and the follow-up visit. A more meaningful contribution could have been made if long-term follow-up had been conducted.

Previous studies have reported major complication rates of 24–27% for Grade IV MPL correction, with acceptable outcomes in 82–93% of cases [15,16,29]. Moreover, achieving a successful prognosis could be more challenging when combining Grade IV MPL repair with the standard TPLO procedure. Therefore, this retrospective case series showed that modified TPLO and TTT were able to successfully correct Grade IV MPL and CCL deficient stifles. Both mTPLO and TPLO-TTT correct quadriceps alignment to a certain degree; therefore, when applied simultaneously, they may induce greater translational change in the quadriceps muscle alignment [10,11,12,13,14]. Considering that MPL is fundamentally a disease caused by the misalignment of the quadriceps femoris muscle, this surgical method can be effectively applied to Grade IV MPL with severe misalignment.

However, concerns have been raised regarding the risk of tibial deformity due to excessive displacement of the quadriceps alignment during this procedure [30]. Although this study did not measure indicators to compare the degree of tibial deformity pre- and post-mTPLO-TTT, previous research using the same modified TPLO plate reported that the degree of deformity was not significant [22]. Additionally, the postoperative clinical gait outcomes were favorable. Thus, an appropriately applied mTPLO-TTT surgical method is unlikely to cause significant tibial deformity.

The implant system used in this study is specifically suitable for small-breed dogs, unlike larger systems reported in earlier studies [31]. Additionally, the mTPLO plate used in this study accommodates 1.5/2.0 mm screws and features a multidirectional locking system, providing the advantage of a fixed-angle locking hole. However, its limitations include the lack of plates with offsets beyond 2 mm, restricting its application in certain cases. Therefore, the development of an mTPLO plate with a 3 or 4 mm offset may be necessary; however, these plates reduce bone contact, thereby impeding bone healing [32]. Therefore, this surgical method may be effective in treating small-breed dogs.

A challenge during surgery was the limited space for multiple implants in small-breed dogs. The pins for TTT were primarily inserted towards the distal tibial fragment to avoid interference with the knee joint, TPLO osteotomy site, and the inserted screws. If interference occurred with the inserted screws, the direction of insertion was adjusted and changed. To prevent the formation of multiple holes in the small tibial plateau fragment, the TTT pin was inserted into the pre-existing holes from the temporary fixation pins used during the TPLO procedure. To minimize pin loosening, a K-wire one size larger than the K-wire used as a temporary fixation pin was employed.

The main limitations of this study include the limited sample size and relatively short follow-up duration. Although the surgical outcomes in this study were excellent, this procedure has inherent structural weaknesses compared with conventional TPLO or medial patellar luxation surgery. Therefore, with an increased number of cases and a longer follow-up period, the complication rate and surgical prognosis may become comparable to or even higher than those reported in other studies. Additionally, small-breed dogs have the advantage of lower physical loading than large-breed dogs. Consequently, if applied to large-breed dogs, the risk of mechanical failure may be higher. However, despite the limited follow-up time, successful bone healing was confirmed. The endpoint, defined as the healing of both the tibial tuberosity and proximal tibial rotational osteotomy, was deemed adequate for excluding major complications and patellar reluxation. To address the limitations of this short-term follow-up, an owner interview was conducted to evaluate the clinical effects beyond 6 months. The results revealed a high level of owner satisfaction with the surgery. However, this study could have been improved if objective clinical examination results were obtained during follow-up visits. Furthermore, although all re-examined dogs exhibited improvements in lameness, the study did not utilize objective lameness assessments because of its retrospective design.

We also acknowledge the potential for long-term complications, such as patellar reluxation, migration of Kirschner wires, irritation from tension band wires, and postoperative infection, which may become apparent with extended monitoring. These complications are known risks associated with TPLO and other surgical techniques used to treat MPL.

In mTPLO-TTT procedures, there is a high risk of fracture at the bone defect site following implant removal. This is similar to secondary fractures that occur at implant removal sites in other fracture repair surgery [33]. Given that the procedure is primarily performed in small-breed dogs with limited space for implant placement, the resulting defect after removal is relatively large compared to the bone size. Therefore, careful consideration of this issue is necessary.

Despite these challenges, this study primarily evaluated the feasibility and clinical outcomes of the mTPLO-TTT technique. Success was characterized by a smooth postoperative recovery, absence of immediate complications, and resolution of MPL in the nine stifles evaluated during follow-up.

## 5. Conclusions

This study demonstrates the feasibility and clinical efficacy of simultaneous mTPLO-TTT for the treatment of concurrent Grade IV MPL and CCLD in small dog breeds. Despite the limited sample size, the results indicate that this approach effectively restores patellar alignment and lameness while minimizing reluxation risk without complications.

While the short-term outcomes are promising, further research involving larger sample sizes and long-term follow-up is essential to validate these findings. Overall, the combined mTPLO-TTT approach represents a viable surgical option for addressing severe MPL and CCLD in small-breeds, contributing to improved outcomes and quality of life for affected patients.

## Figures and Tables

**Table 1:** Radiographic scoring of fracture stability and completeness of fracture healing.

Score	Callus Formation	Fracture Line	Stage of Union
1—Definitely stable	Homogenous	Obliterated	Achieved
2—Probably stable	Massive, trabeculation	Barely discernible	Achieved
3—Questionably stable	Apparent, callus on margins	Discernible	Uncertain
4—Probably unstable	Trace callus, no bridging	Distinct	No
5—Definitely unstable	No callus	Distinct	No

**Table 2:** Details and surgical outcomes of each dog.

Dog	Breed	Weight (kg)	Age (Years)	Lameness Score † (Preoperative)	Lameness Score † (Postoperative)	Follow-Up Time (Weeks)	Radiographic Score ‡
1 left	Maltese	5.5	5	4	2	6	1
1 right	Maltese	5.5	5	5	2	6	2
2	Maltese	3.7	8	4	0	10	1
3	Chihuahua	3.1	6	4	(2) 0	(4) 14	(4) 1
4	Y.T.	3.9	8	4	0	10	1
5	Y.T.	5.2	8	4	0	10	1
6 left	Maltese	3.4	10	4	(2) 0	(6) 13	(3) 1
6 right	Maltese	3.4	10	4	(2) 0	(6) 13	(3) 1
7	Maltese	2.5	12	4	0	10	1

† Lameness scores as provided in text; ‡ radiographic scores as provided in text. Y.T., Yorkshire Terrier. ( ) Additional interim follow-up visit and the corresponding lameness and radiographic scores.

**Table 3:** Owner-assessed outcomes.

Activity Level		Peak Function		Overall Outcome	
Greatly improved	100% (n = 7)	1–2 months	57% (n = 4)	Excellent	100% (n = 7)
Mildly improved		3–4 months	43% (n = 3)	Good	
Same or worse		5–6 months		Fair or poor	

## Data Availability

Data are available in a publicly accessible repository, Data are contained within the article.

## Abbreviations

The following abbreviations are used in this manuscript:

TPLO	tibial plateau leveling osteotomy
mTPLO	modified tibial plateau leveling osteotomy
TPLO-TTT	tibial plateau leveling osteotomy combined with tibial tuberosity transposition
mTPLO-TTT	modified tibial plateau leveling osteotomy and tibial tuberosity transposition
MPL	medial patellar luxation
CCL	cranial cruciate ligament
CCLD	cranial cruciate ligament disease

## References

[1] DeAngelis M. (1971). Patellar luxation in dogs. Vet. Clin. N. Am..

[2] Aragon C.L., Budsberg S.C. (2005). Applications of evidence-based medicine: Cranial cruciate ligament injury repair in the dog. Vet. Surg..

[3] Hayes A.G., Boudrieau R.J., Hungerford L.L. (1994). Frequency and distribution of medial and lateral patellar luxation in dogs: 124 cases (1982–1992). J. Am. Vet. Med. Assoc..

[4] Ness M.G., Abercromby R.H., May C., Turner B.M., Carmichael S. (1996). A survey of orthopaedic conditions in small animal veterinary practice in Britain. Vet. Comp. Orthop. Traumatol..

[5] Willauer C.C., Vasseur P.B. (1987). Clinical results of surgical correction of medial luxation of the patella in dogs. Vet. Surg..

[6] Campbell C.A., Horstman C.L., Mason D.R., Evans R.B. (2010). Severity of patellar luxation and frequency of concomitant cranial cruciate ligament rupture in dogs: 162 cases (2004–2007). J. Am. Vet. Med. Assoc..

[7] L’Eplattenier H., Montavon P. (2002). Patellar luxation in dogs and cats: Pathogenesis and diagnosis. Compendium.

[8] Gibbons S.E., Macias C., Tonzing M.A., Pinchbeck G.L., McKee W.M. (2006). Patellar luxation in 70 large breed dogs. J. Small Anim. Pract..

[9] Witsberger T.H., Villamil J.A., Schultz L.G., Hahn A.W., Cook J.L. (2008). Prevalence of and risk factors for hip dysplasia and cranial cruciate ligament deficiency in dogs. J. Am. Vet. Med. Assoc..

[10] Langenbach A., Marcellin-Little D.J. (2010). Management of concurrent patellar luxation and cranial cruciate ligament rupture using modified tibial plateau levelling. J. Small Anim. Pract..

[11] Flesher K., Beale B.S., Hudson C.C. (2019). Technique and outcome of a modified tibial plateau levelling osteotomy for treatment of concurrent medial patellar luxation and cranial cruciate ligament rupture in 76 stifles. Vet. Comp. Orthop. Traumatol..

[12] Leonard K.C., Kowaleski M.P., Saunders W.B., McCarthy R.J., Boudrieau R.J. (2016). Combined tibial plateau levelling osteotomy and tibial tuberosity transposition for treatment of cranial cruciate ligament insufficiency with concomitant medial patellar luxation. Vet. Comp. Orthop. Traumatol..

[13] Redolfi G., Grand J.-G. (2023). Complications and long-term outcomes after combined tibial plateau leveling osteotomy and tibial tuberosity transposition for treatment of concurrent cranial cruciate ligament rupture and grade III or IV medial patellar luxation. Vet. Comp. Orthop. Traumatol..

[14] Lampart M., Knell S., Pozzi A. (2020). A new approach to treatment selection in dogs with cruciate ligament rupture: Patient-specific treatment recommendations. Schweiz. Arch. Tierheilkd..

[15] Dunlap A.E., Kim S.E., Lewis D.D., Christopher S.A., Pozzi A. (2016). Outcomes and complications following surgical correction of grade IV medial patellar luxation in dogs: 24 cases (2008–2014). J. Am. Vet. Med. Assoc..

[16] Hans E.C., Kerwin S.C., Elliott A.C., Butler R., Saunders W.B., Hulse D.A. (2016). Outcome following surgical correction of grade 4 medial patellar luxation in dogs: 47 stifles (2001–2012). J. Am. Anim. Hosp. Assoc..

[17] Witte P., Scott H. (2011). Investigation of lameness in dogs. Practice.

[18] Duerr F.M. (2020). Canine Lameness.

[19] Krotscheck U., Thompson M.S., Ryan K.K., Mohammed H.O. (2012). Comparison of Tibial PA, bone healing, and intra-articular screw placement using conventional nonlocked application of surgeon-contoured versus locked application of precontoured TPLO plates in dogs. Vet. Surg..

[20] Reif U., Dejardin L.M., Probst C.W., DeCamp C.E., Flo G.L., Johnson A.L. (2004). Influence of limb positioning and measurement method on the magnitude of the tibial plateau angle. Vet. Surg..

[21] Jeon J.-W., Kang K.-W., Kim W.-K., Jung C., Kang B.-J. (2023). Three-dimensional–printed patient-specific guides for tibial deformity correction in small-breed dogs. Am. J. Vet. Res..

[22] Slocum B., Devine T. (1983). Cranial tibial thrust: A primary force in the canine stifle. J. Am. Vet. Med. Assoc..

[23] Jeong E., Jeon Y., Kim T., Lee D., Roh Y. (2024). Assessing the effectiveness of modified tibial plateau leveling osteotomy plates for treating cranial cruciate ligament rupture and medial patellar luxation in small-breed dogs. Animals.

[24] Song Y., Hong Y., Park H., Lee J.-M., Cheong J. (2024). Effects of bupivacaine infiltration anesthesia using temperature-responsive hydrogel in dogs undergoing celiotomy. J. Vet. Clin..

[25] Hammer R.R.R., Hammerby S., Lindholm B. (1985). Accuracy of radiologic assessment of Tibia1 shaft fracture union in humans. Clin. Orthop. Relat. Res..

[26] Gatineau M., Dupuis J., Planté J., Moreau M. (2011). Retrospective study of 476 tibial plateau levelling osteotomy procedures. Vet. Comp. Orthop. Traumatol..

[27] Kennedy K.C., Martinez S.A., Martinez S.E., Tucker R.L., Davies N.M. (2018). Effects of low-level laser therapy on bone healing and signs of pain in dogs following tibial plateau leveling osteotomy. Am. J. Vet. Res..

[28] Knebel J., Eberle D., Steigmeier-Raith S., Reese S., Meyer-Lindenberg A. (2020). Outcome after tibial plateau levelling osteotomy and modified Maquet procedure in dogs with cranial cruciate ligament rupture. Vet. Comp. Orthop. Traumatol..

[29] Wangdee C., Theyse L.F.H., Techakumphu M., Soontornvipart K., Hazewinkel H.A.W. (2013). Evaluation of surgical treatment of medial patellar luxation in Pomeranian dogs. Vet. Comp. Orthop. Traumatol..

[30] Wheeler J.L., Cross A.R., Gingrich W. (2003). In vitro effects of osteotomy angle and osteotomy reduction on tibial angulation and rotation during the tibial plateau-leveling osteotomy procedure. Vet. Surg..

[31] Kloc P.A., Kowaleski M.P., Litsky A.S., Brown N.O., Johnson K.A. (2009). Biomechanical comparison of two alternative tibial plateau leveling osteotomy plates with the original standard in an axially loaded gap model: An in vitro study. Vet. Surg..

[32] Dallago M., Baroncelli A.B., Hudson C., Peirone B., De Bakker E., Piras L.A. (2023). Effect of plate type on tibial plateau levelling and medialization osteotomy for treatment of cranial cruciate ligament rupture and concomitant medial patellar luxation in small breed dogs: An in vitro study. Vet. Comp. Orthop. Traumatol..

[33] Johnson K. (2025). Refracture of the toy breed radius. Vet. Comp. Orthop. Traumatol..

